# Effect of rurality and travel distance on contralateral prophylactic mastectomy for unilateral breast cancer

**DOI:** 10.1007/s10552-023-01689-9

**Published:** 2023-04-25

**Authors:** Madison M. Wahlen, Ingrid M. Lizarraga, Amanda R. Kahl, Whitney E. Zahnd, Jan M. Eberth, Linda Overholser, Natoshia Askelson, Rachel Hirschey, Katherine Yeager, Sarah Nash, Jacklyn M. Engelbart, Mary E. Charlton

**Affiliations:** 1 Department of Epidemiology, University of Iowa, Iowa City, IA USA; 2 Department of Surgery, University of Iowa Hospitals and Clinics, Iowa City, IA USA; 3 Iowa Cancer Registry, Iowa City, IA USA; 4 Department of Health Management and Policy, University of Iowa, Iowa City, IA USA; 5 Department of Epidemiology and Biostatistics, University of South Carolina, Columbia, SC USA; 6 Department of Health Management and Policy, Drexel University, Philadelphia, PA USA; 7 Department of Internal Medicine, University of Colorado School of Medicine, Aurora, CO USA; 8 Department of Community and Behavioral Health, University of Iowa, Iowa City, IA USA; 9 School of Nursing, University of North Carolina, Chapel Hill, NC USA; 10 Nell Hodgson Woodruff School of Nursing, Emory University, Atlanta, GA USA

**Keywords:** Contralateral prophylactic mastectomy, Rurality, Travel distance

## Abstract

**Purpose:**

Despite lack of survival benefit, demand for contralateral prophylactic mastectomy (CPM) to treat unilateral breast cancer remains high. High uptake of CPM has been demonstrated in Midwestern rural women. Greater travel distance for surgical treatment is associated with CPM. Our objective was to examine the relationship between rurality and travel distance to surgery with CPM.

**Methods:**

Women diagnosed with stages I–III unilateral breast cancer between 2007 and 2017 were identified using the National Cancer Database. Logistic regression was used to model likelihood of CPM based on rurality, proximity to metropolitan centers, and travel distance. A multinomial logistic regression model compared factors associated with CPM with reconstruction versus other surgical options.

**Results:**

Both rurality (OR 1.10, 95% CI 1.06–1.15 for non-metro/rural vs. metro) and travel distance (OR 1.37, 95% CI 1.33–1.41 for those who traveled 50 + miles vs. < 30 miles) were independently associated with CPM. For women who traveled 30 + miles, odds of receiving CPM were highest for non-metro/rural women (OR 1.33 for 30–49 miles, OR 1.57 for 50 + miles; reference: metro women traveling < 30 miles). Non-metro/rural women who received reconstruction were more likely to undergo CPM regardless of travel distance (ORs 1.11–1.21). Both metro and metro-adjacent women who received reconstruction were more likely to undergo CPM only if they traveled 30 + miles (ORs 1.24–1.30).

**Conclusion:**

The impact of travel distance on likelihood of CPM varies by patient rurality and receipt of reconstruction. Further research is needed to understand how patient residence, travel burden, and geographic access to comprehensive cancer care services, including reconstruction, influence patient decisions regarding surgery.

**Supplementary Information:**

The online version contains supplementary material available at 10.1007/s10552-023-01689-9.

## Introduction

More than 3.7 million women are living with breast cancer in the United States, and approximately 260,000 more women will be diagnosed in 2022 [1]. For women diagnosed with unilateral breast cancer, unilateral mastectomy (UM) with or without reconstruction or breast conserving therapy (lumpectomy) is the recommended surgical management [2]. Concomitant removal of the unaffected breast, or contralateral prophylactic mastectomy (CPM), may be performed in women with elevated contralateral breast cancer risk such as those with a deleterious genetic mutation (e.g., BRCA 1/2 mutations). The rate of contralateral breast cancer in average-risk women has decreased over the last 20 years and recurrence or survival benefit associated with CPM is lacking [3, 4]. CPM is associated with increased cost, risk of complications, and lower quality of life and, thus, is discouraged by multiple clinical guidelines for women at average-risk [5–8]. Despite this, the rate of CPM for surgical management of breast cancer has continued to increase over the last two decades [3].

Women may elect CPM for several reasons including a desire to decrease anxiety about recurrence, a desire for symmetry after surgery, and to avoid potential additional surgery in the future [9–15]. Characteristics consistently associated with receipt of CPM include young age (< 40 years of age), White race, and social determinants often correlated with affluence such as higher income, education, and private insurance [3, 15–19]. Increased patient autonomy may be another important factor, as CPM is associated with patient-driven decision making and receipt of reconstruction [17]. Together, these factors suggest a patient-demand driven increase in CPM, and that women of higher socioeconomic and educational status with increased access to resources and reconstructive services are most likely to elect and receive CPM.

The social determinants typically associated with CPM, such as higher income, education, and private insurance, are more common among women living in metropolitan areas [20]. Rural communities have less access to high-quality education at all levels, and fewer socioeconomic opportunities which contribute to fewer rural patients with higher education and income and potentially less access to comprehensive healthcare [20, 21]. However, unexpectedly high proportions (42.8–48.5%) of CPM have been reported among women aged 20–44 with early-stage unilateral breast cancer who underwent surgery in Midwestern states that have a high proportion of rural residents. A study in Iowa, which had the second highest proportion of breast cancer patients electing CPM [22], identified the highest rates of CPM among rural-residing women under the age of 40 [23]. Rural patients often travel farther for many aspects of cancer treatment, which contributes to the lower rates of guideline concordant care in this population [24–27]. For example, lack of geographic access to both providers and facilities results in lower rates of chemotherapy, radiation, and reconstruction for rural patients, for whom the burden of travel may be prohibitive for the multiple visits required to complete courses of treatment. [27–29]. Because CPM is considered low-value care in average-risk women, with potential to cause morbidity and adversely impact quality of life, disproportionately high rates of CPM in rural women is concerning and warrants investigation. However, the relationship between rurality and CPM observed in Iowa has not been replicated; indeed, one study using the National Cancer Database (NCDB) did not identify a significant relationship between rurality and receipt of CPM [20, 23].

Given the disagreement in the literature about the association between patient rurality and receipt of CPM, as well as the increased cost, risk of complications, and adverse effect on quality of life associated with receipt of CPM, further investigation into the relationship between rurality and CPM is warranted. In this study, we used data from the NCDB to evaluate whether travel distance, receipt of reconstruction and proximity to urban centers impacted the likelihood of CPM for rural patients receiving breast cancer treatment at Commission on Cancer (CoC) facilities nationwide. We hypothesized that rural patients would be more likely to elect CPM because of longer travel distances in pursuit of access to reconstruction and other multidisciplinary care.

## Methods

### Data source and study population

**Fig. 1:**
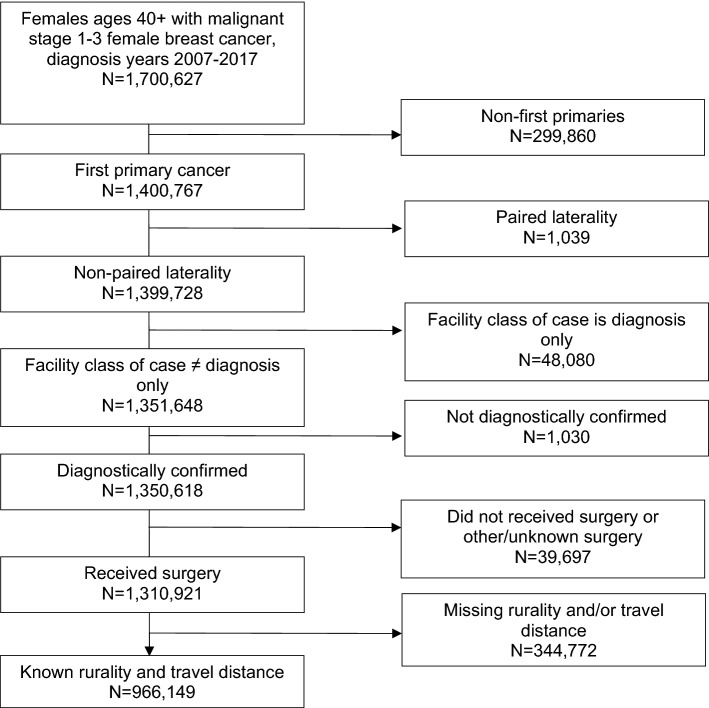
Inclusion criteria

We performed a secondary data analysis using the NCDB, a hospital-based cancer registry system maintained by the American College of Surgeons Commission on Cancer (CoC) [30]. The NCDB includes data from more than 1500 CoC-accredited facilities and represents an estimated 70% of incident cancer cases in the US [30]. We identified a cohort of women aged 40 + years (the NCDB suppresses data on patients < 40 years of age) who were diagnosed with stage I–III unilateral breast cancer between 2007 and 2017 and managed surgically. Cases were excluded from analyses if their cancer was not diagnostically confirmed, they had cancer of bilateral or unknown laterality, their case was only diagnosed and not treated at reporting facility, surgical management was either not performed or unknown, or information about rurality and/or travel distance was unknown (Fig. 1).

### Outcome variable

Surgery type was defined as either UM, CPM, or lumpectomy using the following Standards for Oncology Registry Entry (STORE) codes [31]: UM: 30, 40–41, 43–46, 50–51, 53–56, 61, 64–67, 70–71, and 80; CPM: 42, 47–49, 52, 57–59, 62–63, 68–69, 72–75; lumpectomy: 20–24. Breast reconstruction was defined using the following surgical codes: 43–49, 53–59, 63–69, 73–75.

### Covariates

**Fig. 2:**
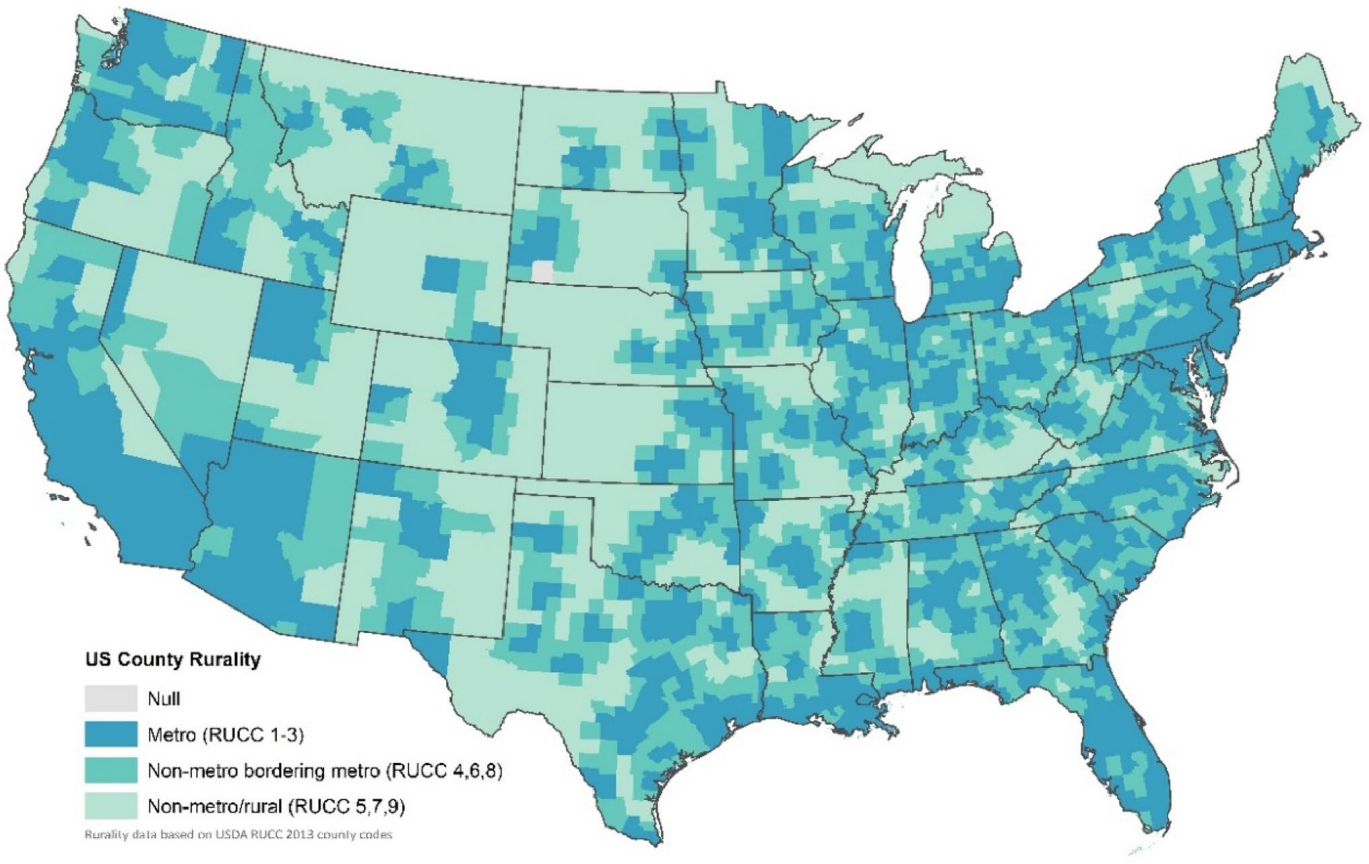
Map of United States counties by rurality classification based on both population and degree of urbanization in adjacent counties

Rurality was defined based on patients’ county of residence using the US Department of Agriculture’s Rural–Urban Continuum Codes (RUCC). For this analysis, we used a categorization of rurality based on both population and degree of urbanization in adjacent counties (Fig. 2). Patients were classified as either metropolitan (metro: RUCC 1–3), non-metro bordering metro (metro-adjacent: RUCC 4, 6, 8), or non-metro not bordering metro (non-metro/rural: RUCC 5, 7, 9). This classification of rurality was used to examine the relationship between patient rurality and CPM from a healthcare delivery standpoint as the two non-metro groups represent decreasing urban adjacency (i.e., likely less access to care) [32]. Distance traveled for surgery was calculated by NCDB and represents the distance in miles between the centroid of patients’ ZIP code and reporting hospital.

Demographic covariates analyzed included age at diagnosis, race/ethnicity, insurance status, area-level income, area-level education, and year of diagnosis. Race and ethnicity were co-categorized as non-Hispanic White (White), non-Hispanic Black (Black), non-Hispanic Asian/Pacific Islander (API), non-Hispanic American Indian/Alaskan Native (AIAN), Hispanic of any race (Hispanic), and non-Hispanic other/unknown (other). We acknowledge that race and ethnicity are social constructs indicative of a potential interpersonal or structural advantage or disadvantage (e.g., racism and discrimination). Therefore, race/ethnicity has been included as a covariate that may affect whether one receives treatment, though not an intrinsic determinant of whether one should receive a specific treatment. Additionally, race/ethnicity may influence patient decision making regarding CPM due to differing sociocultural perspectives and values related to cancer risk and surgical considerations such as symmetry. Income and education were defined using the median income and percent of residents with no high school degree given for each patient’s ZIP code of residence. These were estimated by NCDB by matching the ZIP code of the patient at time of diagnosis to files derived from the 2012 American Community Survey data from 2008 to 2012 using a ZIP to Zip Code Tabulation Area match. Year of diagnosis was included as a covariate to account for potential changes in healthcare delivery and guidelines for breast cancer management across time including the passage of the Affordable Care Act in 2010 and the publication of consensus statements discouraging the use of CPM for average-risk women in 2016 and 2017 [6, 8].

Tumor/clinical variables analyzed included stage at diagnosis, histology, and grade.

Facility variables analyzed included facility type, facility region, whether facility offered reconstruction services, distance traveled for surgery, and average annual surgical volume. Facility types include Community Cancer Programs (facilities with an annual caseload of 100–499 newly diagnosed cancer cases), Comprehensive Community Cancer Programs (annual caseload of 500 + newly diagnosed cancer cases), Academic/Research Program (annual caseload of 500 + newly diagnosed cancer cases, and participate in at least four program areas of postgraduate medical education), and Integrated Network Cancer Programs (facilities that belong to a network of facilities owned by the same organization and offer integrated, comprehensive cancer care services) [30]. A facility was noted to have reconstruction services available if at least one patient included in the analysis was noted to have received reconstruction with their surgery at that facility, using the variable *Surgical Procedure of Primary Site at This Facility* (rx_hosp_surg_prim_site). Average annual surgical volume was calculated by summing the number of patients who received surgery at each hospital per year (2007–2017) and dividing by the number of years the hospital was active. A hospital was noted as active if they saw at least one patient, regardless of surgery, during a year.

### Statistical analysis

Differences in patient demographic, tumor, treatment, and facility characteristics were compared between patient rurality groups and surgery types using Chi-square tests. One way ANOVA was used to compare median travel distance between patient rurality groups. Logistic regression was used to model receipt of CPM based on rurality, travel distance, and their interaction, while adjusting for demographic, tumor, and hospital characteristics. Multinomial logistic regression was used to examine factors associated with CPM with reconstruction compared to unilateral mastectomy with reconstruction, CPM without reconstruction, and unilateral mastectomy/lumpectomy without reconstruction. Complete case analysis was used to construct our models. No patients were coded as receiving lumpectomy with reconstruction in our dataset. Analyses were conducted using SAS 9.4 (SAS Institute, Cary, NC).

## Results

A total of 966,149 women were included in this analysis. Most of the women were white (79%), above the age of 50 (82%), lived in metropolitan areas (87%) and had private insurance (52%) (Table 1). More than half of the women had stage I disease at diagnosis (57%), and most received lumpectomy (60%) while 11% received CPM for breast cancer treatment.

**Table 1:** Demographics, tumor, treatment, and hospital characteristics for female breast cancer cases by patient rurality, 2007–2017

	All		Patient Rurality						Chi sq *p*-value
			Metro		Metro-adjacent		Non-metro/Rural		
	*n*	Col %	*n*	Col %	*n*	Col %	*n*	Col %	
All	966,149	100%	841,087	87%	88,016	9%	37,046	4%	
Age at Diagnosis									
40–49 years	172,568	18%	153,651	18%	13,416	15%	5,501	15%	** < 0.0001**
50–59 years	253,039	26%	221,549	26%	22,151	25%	9,339	25%	
60–69 years	274,281	28%	236,214	28%	26,852	31%	11,215	30%	
70 + years	266,261	28%	229,673	27%	25,597	29%	10,991	30%	
Race/Ethnicity									
American Indian	2,537	0%	1,606	0%	537	1%	394	1%	** < 0.0001**
Asian/Pacific Islander	34,227	4%	33,435	4%	355	0%	437	1%	
Black	105,405	11%	97,336	12%	6,492	7%	1,577	4%	
Hispanic	53,452	6%	51,392	6%	1,373	2%	687	2%	
Other/Unknown	12,117	1%	11,367	1%	506	1%	244	1%	
White	758,411	79%	645,951	77%	78,753	89%	33,707	91%	
Insurance									
Not Insured	17,181	2%	15,040	2%	1,524	2%	617	2%	** < 0.0001**
Private Insurance	501,578	52%	444,409	53%	40,607	46%	16,562	45%	
Medicaid	57,974	6%	50,539	6%	5,158	6%	2,277	6%	
Medicare	366,777	38%	311,557	37%	38,562	44%	16,658	45%	
Other Government	8,895	1%	7,366	1%	1,020	1%	509	1%	
Insurance Status Unknown	13,744	1%	12,176	1%	1,145	1%	423	1%	
Year of Diagnosis									
2007–2009	244,265	25%	210,299	25%	23,823	27%	10,143	27%	** < 0.0001**
2010–2013	353,056	37%	308,687	37%	31,182	35%	13,187	36%	
2014–2017	368,828	38%	322,101	38%	33,011	38%	13,716	37%	
Median Income Quartiles 2008–2012									
Missing	885		658		135		92		** < 0.0001**
< $38,000	142,499	15%	102,796	12%	27,060	31%	12,643	34%	
$38,000-$47,999	202,817	21%	152,165	18%	36,345	41%	14,307	39%	
$48,000-$62,999	257,967	27%	229,936	27%	19,615	22%	8,416	23%	
≥ $63,000	361,981	38%	355,532	42%	4,861	6%	1,588	4%	
Percent No High School Degree Quartiles 2008–2012									
Missing	548		409		80		59		** < 0.0001**
≥ 21%	144,815	15%	114,141	14%	22,164	25%	8,510	23%	
13.0–20.9%	227,660	24%	184,085	22%	33,001	38%	10,574	29%	
7.0–12.9%	319,172	33%	280,102	33%	25,257	29%	13,813	37%	
< 7.0%	273,954	28%	262,350	31%	7,514	9%	4,090	11%	
Stage at diagnosis									
1	546,912	57%	477,787	57%	48,710	55%	20,415	55%	** < 0.0001**
2	320,608	33%	278,214	33%	29,943	34%	12,451	34%	
3	98,629	10%	85,086	10%	9,363	11%	4,180	11%	
Histology									
Invasive ductal carcinoma	731,151	76%	635,025	76%	67,731	77%	28,395	77%	** < 0.0001**
Invasive lobular carcinoma	180,925	19%	159,591	19%	15,036	17%	6,298	17%	
Other	54,073	6%	46,471	6%	5,249	6%	2,353	6%	
Grade									
1	225,800	23%	195,420	23%	21,010	24%	9,370	25%	** < 0.0001**
2	413,247	43%	360,988	43%	36,929	42%	15,330	41%	
3	277,263	29%	241,388	29%	25,383	29%	10,492	28%	
4	2,078	0%	1,739	0%	224	0%	115	0%	
Unknown	47,761	5%	41,552	5%	4,470	5%	1,739	5%	
Surgery type									
CPM	111,030	11%	96,027	11%	10,220	12%	4,783	13%	** < 0.0001**
Lumpectomy	575,722	60%	505,949	60%	49,845	57%	19,928	54%	
Unilateral mastectomy	279,397	29%	239,111	28%	27,951	32%	12,335	33%	
Surgery type and reconstruction									
CPM and no reconstruction	49,000	5%	40,571	5%	5,683	6%	2,746	7%	** < 0.0001**
CPM and reconstruction	62,030	6%	55,456	7%	4,537	5%	2,037	6%	
Lumpectomy	575,722	60%	505,949	60%	49,845	57%	19,928	54%	
ULM and no reconstruction	216,181	22%	181,846	22%	23,716	27%	10,619	29%	
ULM and reconstruction	63,216	7%	57,265	7%	4,235	5%	1,716	5%	
Distance traveled to surgery hospital (miles)									
< 30 miles	842,954	87%	784,255	93%	44,574	51%	14,125	38%	** < 0.0001**
30–49 mil	63,525	7%	32,956	4%	25,207	29%	5,362	14%	
50 + miles	59,670	6%	23,876	3%	18,235	21%	17,559	47%	
Facility type									
Community Cancer Program	95,124	10%	69,550	8%	16,139	18%	9,435	25%	** < 0.0001**
Comprehensive Community Cancer Program	452,740	47%	388,553	46%	45,470	52%	18,717	51%	
Academic/Research Program	288,005	30%	262,752	31%	17,951	20%	7,302	20%	
Integrated Network Cancer Program	130,280	13%	120,232	14%	8,456	10%	1,592	4%	
Facility region									
Midwest	238,667	25%	193,295	23%	29,459	33%	15,913	43%	** < 0.0001**
Northeast	203,944	21%	190,728	23%	11,352	13%	1,864	5%	
South	360,518	37%	306,473	36%	41,189	47%	12,856	35%	
West	163,020	17%	150,591	18%	6,016	7%	6,413	17%	
Facility has reconstruction services									
No	9,040	1%	3,504	0%	4,058	5%	1,478	4%	** < 0.0001**
Yes	957,109	99%	837,583	100%	83,958	95%	35,568	96%	
Average annual surgical volume 2007–2017									
< 50	61,204	6%	42,875	5%	12,714	14%	5,615	15%	** < 0.0001**
50–99	153,050	16%	125,647	15%	17,109	19%	10,294	28%	
100–174	210,021	22%	182,175	22%	20,870	24%	6,976	19%	
175 +	541,874	56%	490,390	58%	37,323	42%	14,161	38%	

Compared to those in non-metro areas, a higher proportion of metro patients were younger (40–49 years), non-white, had private insurance and lived in a ZIP code with a higher median income and lower percentage of people without a high school degree (Table 1). A higher proportion of non-metro/rural patients were older, white, had Medicare, and lived in a ZIP code with a median income less than $47,999 and where more than 13% of the population did not have a high school degree. Non-metro/rural and metro-adjacent patients had similar demographic and clinical characteristics, though more non-metro/rural patients lived in a ZIP code with the lowest income quartile (34% vs. 31%). Sixty-three percent of the metro-adjacent cohort lived in a ZIP code where more than 13% of the population did not have high school degrees.

Differences in treatment type by rurality are also given in Table 1. Metro patients had the highest proportion of lumpectomy and were also more likely to receive reconstruction with mastectomy. In contrast, rural women had a slightly higher proportion of CPM, and underwent CPMs without reconstruction more than those in metro areas. Non-metro/rural women had the highest proportion of CPM in every age group (data not shown).

There were also differences in distance traveled to care. Median travel distance was 8 miles (IQR 4–14 miles) for metro patients, 30 miles (IQR 17–46 miles) for metro-adjacent patients, and 47 miles (IQR 16–81 miles) for non-metro/rural patients. In terms of hospitals characteristics, almost half of non-metro/rural patients were treated at facilities with an annual surgical volume under 100 cases/year (43%), compared to 33% of metro-adjacent and 20% of metro patients. Comprehensive Community Cancer Programs were the most common surgery facility for all patients, regardless of rurality. Metro patients received surgery more often at academic/research programs, while non-metro/rural patients more often went to Community Cancer Programs (25% vs. 18% metro-adjacent and 8% metro).

**Fig. 3:**
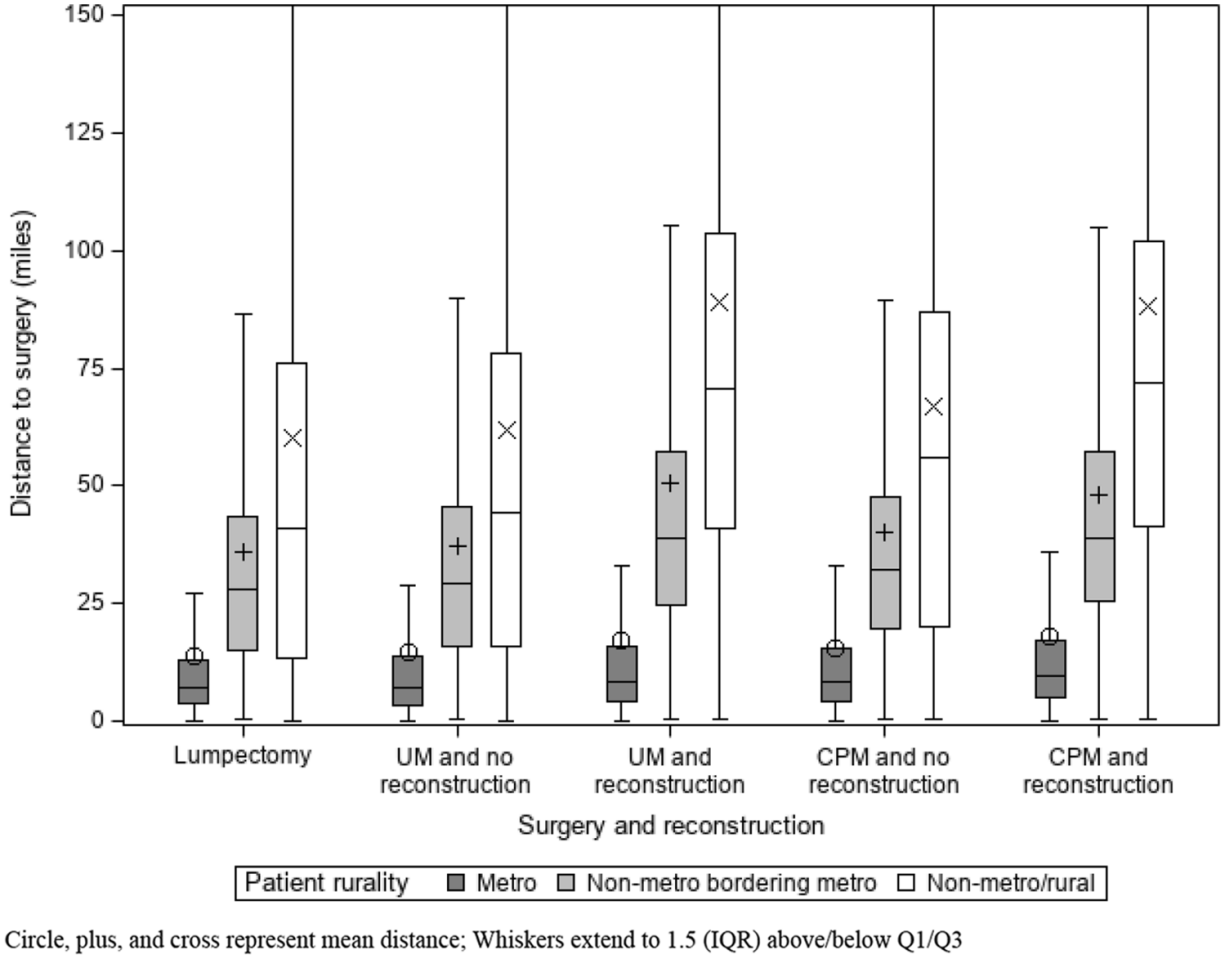
Median distance traveled for surgery (with/without reconstruction) for stage 0-III breast cancer by patient residence. Circle, plus, and cross represent mean distance; Whiskers extend to 1.5 (IQR) above/below Q1/Q3

Figure 3 demonstrates how the median travel distance to surgery facility varied by patient rurality. There were significant differences in travel distance between rurality groups by surgery type and receipt of reconstruction. Metro patients had little variation in median travel distance (7–10 miles), regardless of surgery type. Those who received reconstruction traveled further (metro-adjacent: 39 miles CPM, 39 miles UM; non-metro/rural: 72 miles Cm, was associated with low PM, 71 miles UM) than those who did not (metro-adjacent: 32 miles CPM, 30 miles UM; non-metro/rural: 56 miles CPM, 45 miles UM). The median travel distance for a lumpectomy was similar to the median travel distance for a UM without reconstruction (metro-adjacent: 28 miles; non-metro/rural: 41 miles).

### Multivariable model

Adjusting for demographic, tumor, and hospital characteristics, we observed higher odds of receiving a CPM for both women who traveled a greater distance for surgical care (OR 1.37 for those who traveled 50 + miles vs. those who traveled < 30 miles, 95% CI 1.33–1.41), and for those who lived in non-metro/rural counties (OR 1.10 for non-metro/rural compared to metro, 95% CI 1.06–1.15) (data not shown).

Table 2 indicates multivariable-adjusted odds of receiving CPM, showing interactions for rurality/distance traveled to care, as well as covariates. Regardless of patient rurality, the odds of receiving a CPM were significantly higher for all women who traveled at least 30 miles for surgery compared to metro women traveling less than 30 miles. Among those who traveled more than 30 miles, odds of receiving CPM were highest for non-metro/rural women (OR 1.33, 95% CI 1.22–1.45 for those who traveled 30–49 miles; OR 1.57, 95% CI 1.50–1.64 for those who traveled 50 + miles).

**Table 2:** Multivariable-adjusted logistic regression model: Odds of receiving CPM

	OR	CI
Interaction term: Patient residence × travel distance to surgery facility		
Metro patient who traveled < 30 miles	1.00	REF
Non-metro bordering metro patient who traveled < 30 miles	1.00	(0.96, 1.03)
Non-metro/Rural patient who traveled < 30 miles	1.05	(0.99, 1.12)
Metro patient who traveled 30–49 miles	**1.30**	**(1.26, 1.34)**
Non-metro bordering metro patient who traveled 30–49 miles	**1.28**	**(1.23, 1.33)**
Non-metro/Rural patient who traveled 30–49 miles	**1.33**	**(1.22, 1.45)**
Metro patient who traveled 50 + miles	**1.33**	**(1.28, 1.38)**
Non-metro bordering metro patient who traveled 50 + miles	**1.38**	**(1.32, 1.44)**
Non-metro/Rural patient who traveled 50 + miles	**1.57**	**(1.50, 1.64)**
Demographic characteristics		
Age at Diagnosis		
40–49 years	**7.62**	**(7.39, 7.85)**
50–59 years	**3.98**	**(3.86, 4.09)**
60–69 years	**2.37**	**(2.31, 2.43)**
70 + years	1.00	REF
Race/Ethnicity		
White	1.00	REF
American Indian	**0.67**	**(0.59, 0.77)**
Asian/Pacific Islander	**0.55**	**(0.52, 0.57)**
Black	**0.54**	**(0.53, 0.55)**
Hispanic	**0.69**	**(0.66, 0.71)**
Other/Unknown	**0.79**	**(0.74, 0.84)**
Insurance		
Private Insurance	1.00	REF
Not Insured	**0.56**	**(0.53, 0.59)**
Medicaid	**0.73**	**(0.71, 0.75)**
Medicare	**0.80**	**(0.79, 0.82)**
Other government	0.99	(0.93, 1.05)
Insurance status unknown	**0.44**	**(0.41, 0.47)**
Year of diagnosis		
2007–2009	**0.59**	**(0.58, 0.60)**
2010–2013	**0.91**	**(0.90, 0.93)**
2014–2017	1.00	REF
Median Income Quartiles 2008–2012		
< $38,000	**0.94**	**(0.91, 0.97)**
$38,000-$47,999	0.97	(0.95, 1.00)
$48,000-$62,999	1.00	(0.99, 1.02)
≥ $63,000	1.00	REF
Percent No High School Degree Quartiles 2008–2012		
≥ 21%	**0.79**	**(0.76, 0.81)**
13.0–20.9%	**0.85**	**(0.83, 0.87)**
7.0–12.9%	**0.92**	**(0.91, 0.94)**
< 7.0%	1.00	REF
Tumor characteristics		
Stage at Diagnosis		
1	1.00	REF
2	**1.45**	**(1.43, 1.47)**
3	**1.93**	**(1.89, 1.97)**
Histology		
Invasive ductal carcinoma	1.00	REF
Invasive lobular carcinoma	**1.38**	**(1.35, 1.40)**
Other	**0.93**	**(0.90, 0.96)**
Grade		
Well differentiated	1.00	REF
Moderately differentiated	**1.18**	**(1.16, 1.21)**
Poorly differentiated	**1.38**	**(1.36, 1.41)**
Undifferentiated, anaplastic	**1.36**	**(1.19, 1.56)**
Unknown	**1.39**	**(1.35, 1.44)**
Hospital characteristics		
Facility Region		
Northeast	1.00	REF
Midwest	**1.32**	**(1.29, 1.35)**
South	**1.63**	**(1.59, 1.66)**
West	**1.45**	**(1.41, 1.48)**
Facility Type		
Comprehensive Community Cancer Program	1.00	REF
Community Cancer Program	**0.89**	**(0.86, 0.92)**
Academic/Research Program	**0.89**	**(0.88, 0.91)**
Integrated Network Cancer Program	**1.10**	**(1.08, 1.12)**
Surgery facility had reconstruction services		
Yes	1.00	REF
No	**0.51**	**(0.45, 0.56)**
Average annual breast surgical volume 2007–2017		
< 50	**0.77**	**(0.74, 0.80)**
50–99	**0.80**	**(0.78, 0.82)**
100–174	**0.97**	**(0.96, 0.99)**
175 +	1.00	REF

Bold text indicates statistically significant estimates

We also examined associations of demographic, clinical, and facility-level factors with receipt of CPM. The highest odds of CPM were seen in women who were younger (OR 7.62 for women 40–49 years vs. women 70 + years, 95% CI 7.39–7.85). There was decreased odds of CPM for those without insurance (OR 0.56 compared to those with private insurance, 95% CI 0.53–0.59), and those from ZIP codes with the lowest income and education (OR 0.94 for lowest vs. highest income quartile; OR 0.79 for those in the lowest vs. highest education quartile). Hospital characteristics associated with CPM included Integrated Network Cancer Program category (OR 1.10 compared to Comprehensive Community Cancer Program, 95% CI 1.08–1.12), lower surgical volume (OR 0.77 for facilities with an average annual breast surgical volume of < 50 compared to 175 +, 95% CI 0.74–0.80) and availability of reconstruction services (OR 0.51 for facilities not offering reconstructive services compared to those offering reconstructive services, 95% CI 0.45–0.56). Treatment at an academic/research program or a Community Cancer Program were both associated with decreased odds of receiving CPM compared to a comprehensive community cancer program. Odds of CPM were higher in all regions compared to the Northeast, with the highest OR seen in the South (OR 1.63, 95% CI 1.59–1.66). CPM was also associated with higher stage, higher grade, and invasive lobular histology (compared to invasive ductal carcinoma).

### Multinomial model

**Fig. 4:**
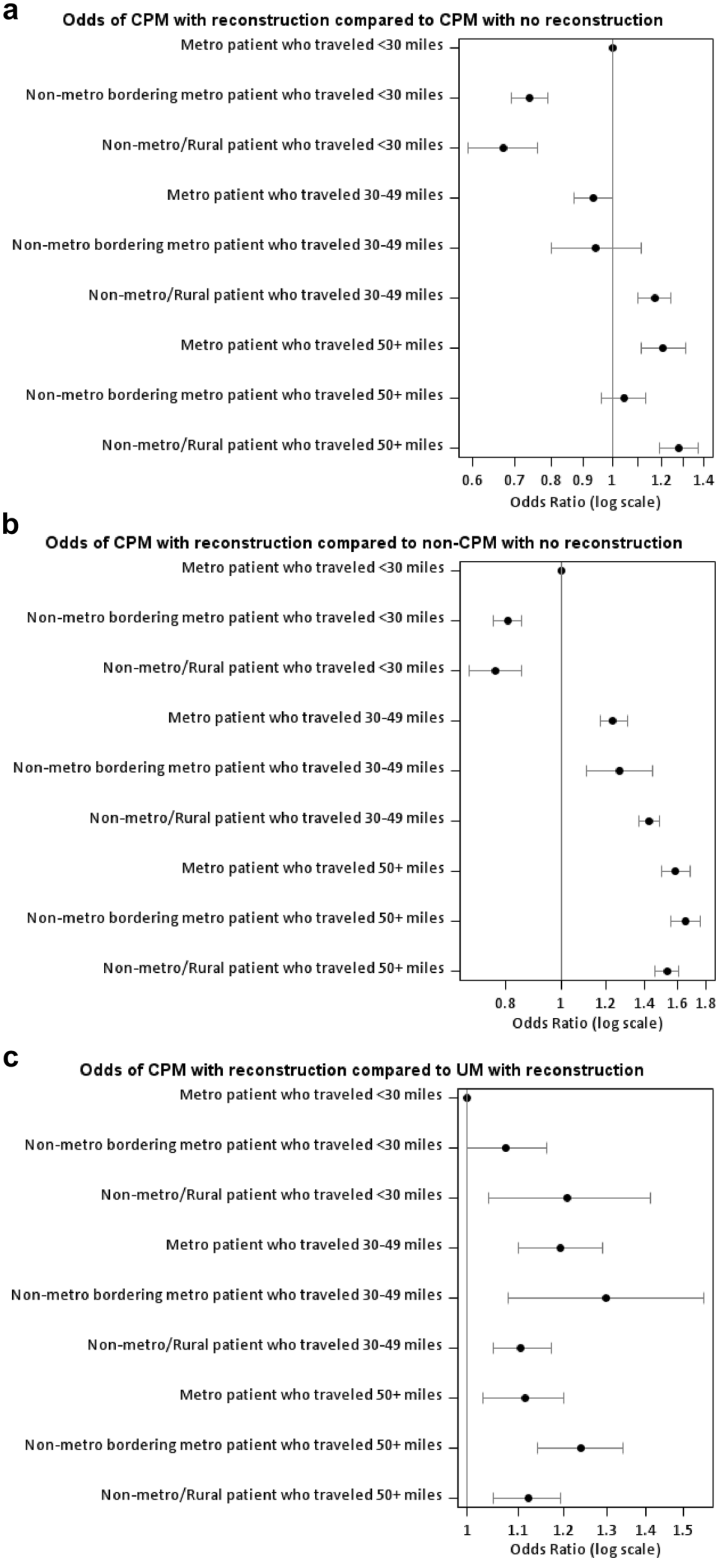
Forest plots comparing odds of CPM with reconstruction with alternative surgical options. **a** Odds of CPM with reconstruction compared to CPM with no reconstruction. **b** Odds of CPM with reconstruction compared to non-CPM with no reconstruction. Non-CPM with no reconstruction includes UM with no reconstruction (27%) and lumpectomies (73%). **c** Odds of CPM with reconstruction compared to UM with reconstruction. Multinomial model adjusted for age at diagnosis, race/ethnicity, insurance, year of diagnosis, median income quartiles, percent no high school degree, stage at diagnosis, histology, grade, facility type, facility region, availability of reconstructive surgery, and average annual surgical volume

Because access to and desire for reconstruction is strongly associated with receipt of CPM, we performed multinomial logistic regression using receipt of CPM with reconstruction as the reference against other possible surgical options (CPM without reconstruction, UM with reconstruction and unilateral breast surgery without reconstruction (includes lumpectomy and UM without reconstruction)) (Fig. 4; Supplementary Table 1). Metro patients traveling < 30 miles for surgery were the reference group for all comparisons across surgery groups. The odds of CPM with reconstruction compared to unilateral breast surgery without reconstruction were lower for all non-metro women who receive surgery within 30 miles (OR 0.81, 95% CI 0.76–0.85 for metro-adjacent and OR 0.77, 95% CI 0.69–0.85 for non-metro/rural), but higher for any patient who traveled beyond that distance, with OR higher for longer travel distances (1.23–1.43 for 30–49 miles and 1.54–1.65 for 50 + miles) but varying little with rurality.

Among women who received reconstruction, non-metro/rural women were more likely to undergo CPM regardless of travel distance (OR 1.11–1.21), while both metro and metro-adjacent women were more likely to undergo CPM only if they traveled 30 + miles (OR 1.24–1.30).

Among women who elected CPM, both non-metro/rural and metro-adjacent were less likely than metro women to receive reconstruction if they received surgery within 30 miles (OR 0.74 95% CI 0.69–0.79 and OR 0.67 95% CI 0.59–0.76 respectively). However, for those women who traveled > 30 miles, likelihood of reconstruction was higher for non-metro/rural women regardless of travel distance (OR 1.17–1.28), and for metro women who traveled > 50 miles (OR 1.21 95% CI 1.11–1.31).

## Discussion

In this study, we analyzed NCDB data to examine associations of CPM with rurality and distance to treatment, adjusted for demographic, clinical, and facility factors. Our analysis demonstrated that residence in a non-metro/rural county was associated with the highest proportion of CPM in women treated for breast cancer at CoC hospitals, compared to residence in a metro or metro-adjacent county. This finding was unexpected and has not been previously demonstrated in a large national database. We also observed associations of CPM with younger age and measures of affluence, including private insurance, and higher income and education, all of which have been well documented in multiple datasets [3, 15–19]. In our study, rural patients in the NCDB were much less likely to exhibit these characteristics than metro patients. Nevertheless, we found an independent association between residence in rural areas that were remote from urban centers and receipt of CPM after adjustment for the more well-established factors of age, education, income, and insurance. Thus, there must conceivably be an alternative mechanism contributing to the increased odds of CPM among rural women, which is concerning since CPM is not indicated for most average-risk women and can be associated with adverse outcomes.

The relationship between rurality and receipt of CPM was partially explained by travel distance, which we found impacted the likelihood of CPM for all patients regardless of rurality. The results of these analyses suggest that the definition and type of rurality is especially important when exploring relationships between patient rurality and clinical outcomes. An NCDB study examining surgical management among breast cancer patients demonstrated no relationship between CPM and patient rurality when classifying rurality using population size categories alone, without consideration of adjacency to metropolitan areas or travel distance [17]. Our results likely differed because we utilized a categorization of rurality based on both population and adjacency to metropolitan counties to examine the relationship between patient rurality and CPM. We anticipated that those living in non-metro counties adjacent to metro counties have increased access to care through shorter commutes to high-volume centers typically located in metro areas, compared to those living in non-metro/non-adjacent counties [32]. Our results support this conclusion, as patients from non-metro/rural counties traveled almost twice as far for surgical treatment as patients from metro-adjacent counties and were more likely to be treated at smaller hospitals with lower case volumes.

However, the relationship between travel distance and rurality is complex. The association between increased travel distance and higher likelihood of CPM has been previously studied in the NCDB, with Ward et al. finding that travel distance had the most significant impact on surgery choice for Black and Hispanic patients [19]. We similarly demonstrate that although traveling farther for surgery appears to increase the likelihood of CPM for all patients, the impact of distance is most significant for non-metro/rural patients. These are also the patients most affected by distance, with almost half of patients living in non-metro/rural counties traveling 50 + miles compared to 21% of those living in metro-adjacent or 3% in metro counties. It is possible that the institutions most frequented by rural patients are not providing the same degree of counseling on the risks and benefits of CPM, perhaps because of limited personnel and resources at those facilities. However, the relationship between CPM and rurality persists even after accounting for facility type, and the most common surgery facility type for rural patients, the Community Cancer Program, was associated with lower rates of CPM than others, suggesting that differences in treating facility is unlikely to be the main driver of the observed trend. It is also possible that the standard format of CPM counseling does not resonate with this patient population. CPM decision aids do exist but largely focus on intrinsic values and knowledge and may not consider extrinsic factors related to resources and access to care, which may be more important to minority and rural women [33–35]. Non-metro/rural women receiving reconstruction traveled over 70 miles on average for surgery, almost twice as far as those from metro-adjacent counties. Breast cancer surgery often necessitates multiple visits preoperatively and the post-operative course and subsequent surveillance can also require frequent face-to face encounters. Women facing a high travel burden may perceive CPM as one way to minimize future need for healthcare utilization, such as radiation therapy and mammograms, especially if there are limited resources for travel.

We used a multinomial model to further explore the relationship between CPM and rurality, travel distance, and reconstruction. Type of surgery was impacted differently by distance traveled depending on patient residence. For those traveling < 30 miles, both non-metro/ rural and metro-adjacent patients were more likely to have lumpectomy or unilateral mastectomy without reconstruction than CPM with reconstruction when compared to metro patients. These findings may represent lack of access to reconstruction in areas proximate to non-metro counties, or a lack of suitability or inclination for reconstruction in non-metro women who elect not to or are unable to travel farther. While we cannot determine from this dataset whether the distance traveled by the patient represents the distance to the nearest available treating facility, one study using Iowa Cancer Registry data found that rural women frequently traveled to large tertiary care centers for surgery, which were often not the nearest hospitals offering breast cancer surgery [23]. In that study, rural women traveling to large tertiary facilities were more likely to elect CPM and have reconstruction than metro women treated in similar facilities [23]. Previous analyses of the NCDB have found that women undergoing reconstruction travel farther than those who do not, and that access to reconstruction may partly explain the link between longer travel distance and CPM [36]. There is a strong association between use of CPM and receipt of breast reconstruction, although the reasons for this are not fully elucidated [37]. In contrast, for women who did receive reconstruction in our analysis, patient rurality more strongly impacted the likelihood of CPM than travel distance. Non-metro/rural patients who received reconstruction were more likely to elect CPM than unilateral mastectomy regardless of travel distance compared to metro patients who stay close to home for treatment (93% of all metro patients). Although the effect size is relatively small, this finding is significant as rurality has not been previously identified as an independent contributor to CPM when travel distance and reconstruction are accounted for. We postulate that even for those non-metro/rural patients with a shorter travel distance, access to reconstruction may require driving to hospitals in a different community. It is unknown how access to care impacts patients’ surgical choices; however, desire to limit future imaging or surgery to achieve symmetry post-reconstruction has been cited as reasons for CPM and may be even more pertinent for patients with a higher travel burden [9–15].

Although CPM is often a patient-driven phenomenon, physician counseling has been shown to have an important role in determining whether the patient ultimately undergoes that operation [18, 38]. We identified variability in practice patterns across the country, by surgical volume and CoC program category. In general, patients were more likely to undergo CPM at larger facilities with reconstructive capabilities. It is unclear whether this is because patients desiring CPM seek out institutions that offer reconstruction, or because of factors specific to the institution. Higher stage and grade were both associated with CPM on multivariable analysis, as was lobular cancer. Lobular carcinoma is not an indication for CPM and prophylactic surgery would provide even less benefit than usual in more aggressive, higher stage disease [7] so it is possible that patients are being recommended CPM inappropriately or are being incompletely counseled about risks and benefits of the surgery. Treatment at an academic/research program was associated with a lower likelihood of CPM, which may reflect a greater awareness in these settings of literature about the rising rate and pitfalls of CPM as well as consensus statements published in 2016 and 2017 discouraging the use of CPM in average-risk women [6–8]. Non-metro women were far less likely to be treated at academic/research programs than those with metro residences. Rural patients may benefit from more widespread provider education regarding the risks and recommendations regarding CPM.

As CPM has not been proven to provide a survival benefit among average-risk women, it is important to consider whether our finding that rural women are more likely to undergo CPM represents a true health disparity. In general, it appears that rural women who travel the furthest distances or who can access reconstructive services are the most likely to undergo CPM. In at least one state, rural women who traveled longer distances to receive CPM were younger, had private insurance, and were from more affluent and educated rural counties than their rural counterparts who did not undergo CPM, suggesting that they had more resources to travel to seek more comprehensive cancer services [23]. With this context and the association between CPM and indicators of affluence in our study and others [17], it seems unlikely that those rural women with the most social and economic disadvantages are the ones who undergo CPM. However, it does appear that travel burden and limited access to high-quality cancer care can impact the surgical choices made by rural women, and this disproportionately affects those women living in rural communities that are remote from more populous areas with robust healthcare resources. Because CPM in average-risk women does not decrease cancer recurrence risk or improve survival and has significant potential adverse effects for patients, including increased cost, risk of complications and poorer quality of life, the possibility that access to care is impacting a woman’s decision to undergo CPM is concerning and warrants further investigation and intervention. Qualitative studies examining the role of travel burden and access to care in surgical decision making may help contextualize our understanding of these factors as decision-making factors among rural women electing CPM. Among rural women with gynecologic cancers, for example, one qualitative study found that recommendations from physicians and others were the primary drivers of travel, despite the associated burdens [39]. Studies like this among rural women electing CPM may help identify intervention strategies to reduce guideline non-concordant CPM among this population.

A major strength of our study is the classification of rurality as a measure of both population size and adjacency to metropolitan counties, as this allows for more meaningful interpretations of results regarding access to care. The use of the NCDB is an additional strength, as it captures an estimated 70% of all cancer cases nationally, permitting analyses of a large sample of breast cancer cases across the country [30]. However, the use of the NCDB is also a limitation of the study, as it is a hospital-based registry system collecting data only from CoC-accredited facilities. Currently only 16% of non-metro/rural hospitals are CoC-accredited [40] and rural patients are underrepresented in the NCDB [20]. Previous work suggests that at least in one state, CoC accreditation is associated with a lower likelihood of CPM and so using the NCDB may underestimate the impact of rurality by limiting analysis only to the highly selected group of rural patients treated at CoC hospitals [23]. Missing data is another important limitation of the NCDB, as more than 300,000 women were excluded from our analyses due to missing information about patient rurality and/or travel distance. This does have the potential to bias the results as the excluded patients had a slightly lower rate of CPM (9% vs 11%). Furthermore, the NCDB does not collect information about other factors which may impact surgical decision making such as genetic testing, family history, use of MRI, patient decision-making preference or surgeon counseling [9, 41–43]. This is an important limitation of our study, as factors such as family history and deleterious mutation carrier status may increase contralateral breast cancer risk enough to confer consensus-concordant receipt of CPM [7]. Thus, it is likely that some of the women in our sample received CPM appropriately, although only 5–10% of breast cancer cases are caused by rare deleterious mutations such as BRCA 1 and 2 [44]. Additionally, NCDB data does not allow us to assess the interaction between hospital rurality and patient rurality. Finally, because the CoC suppresses hospital data for patients aged < 40 years, we had to limit our analysis to women aged > 40 years. Although women aged under 40 years represented a very small proportion of our original sample (5.6%), this is a major limitation as women under 40 represent the demographic with the highest rates of CPM, and previous work outside the NCDB suggests that this age group has the largest differences in CPM based on rurality [23].

## Conclusion

Patient rurality and travel distance are independently associated with CPM. Women living in more remote rural communities are disproportionately affected by travel burden when seeking care at CoC-accredited hospitals, and those with the resources to travel further to access reconstruction and other services are more likely to elect CPM. Supporting high-quality cancer care in non-metro centers and developing patient navigation strategies that mitigate the socioeconomic burden of travel may reduce access to care as a determinant of CPM. Improving access to both reconstruction and multidisciplinary guideline concordant breast cancer treatment for these rural patients will require working collaboratively with rural and rural adjacent hospitals to develop these services. Our findings also support provider education at all CoC institutions on the determinants, risks, and current recommendations about CPM. Qualitative studies of rural patients electing more extensive surgery are needed to better understand how residence and travel distance impact patient decision making and can help to inform more effective patient centered counseling strategies.

### Supplementary Information

Below is the link to the electronic supplementary material.

Supplementary file1 (DOCX 27 kb)

## Data Availability

The datasets generated during and/or analyzed during the current study are available through an application process from the National Cancer Database Participant User Files: https://www.facs.org/quality-programs/cancer-programs/national-cancer-database/puf/.
