# Supplementary material for: Effect of rurality and travel distance on contralateral prophylactic mastectomy for unilateral breast cancer
Source: Cancer Causes Control. 2023 Apr 25;34(Suppl 1):171–86. doi: 10.1007/s10552-023-01689-9 (PMC10689552; doi:10.1007/s10552-023-01689-9)
Supplement: Supplementary file 1 — Supplementary file1 (DOCX 27 kb) [file 10552_2023_1689_MOESM1_ESM.docx]

**Title:** Effect of Rurality and Travel Distance on Contralateral Prophylactic Mastectomy for Unilateral Breast Cancer

**Journal:** *Cancer Causes & Control*

**Authors:** Madison M Wahlen, BS^1^; Ingrid M Lizarraga, MBBS^2^; Amanda R Kahl, MPH^3^; Whitney E Zahnd, PhD^4^, Jan M Eberth, PhD^5,6^; Linda Overholser, MD, MPH^7^; Natoshia Askelson, PhD^8^; Rachel Hirschey, PhD, RN^9^; Katherine Yeager, PhD^10^, RN; Sarah Nash, PhD^1,3^; Jacklyn M Engelbart, MD^2^; Mary E Charlton, PhD^1,3^

^1^University of Iowa, Department of Epidemiology, Iowa City, IA, USA

^2^University of Iowa Hospitals and Clinics, Department of Surgery, Iowa City, IA, USA

^3^Iowa Cancer Registry, Iowa City, IA, USA

^4^University of Iowa, Department of Health Management and Policy, Iowa City, IA, USA

^5^University of South Carolina, Department of Epidemiology and Biostatistics, Columbia, SC, USA

^6^Drexel University, Department of Health Management and Policy, Philadelphia, PA, USA

^7^University of Colorado School of Medicine, Department of Internal Medicine, Aurora, CO, USA

^8^University of Iowa, Department of Community and Behavioral Health, Iowa City, IA, USA

^9^University of North Carolina, School of Nursing, Chapel Hill, NC, USA

^10^Emory University, Nell Hodgson Woodruff School of Nursing, Atlanta, GA, USA

**Corresponding Author:**

Ingrid M Lizarraga, MBBS

[Ingrid-lizarraga@uiowa.edu](mailto:Ingrid-lizarraga@uiowa.edu)

Supplementary Table 1. Multinomial model odds of receiving CPM reconstruction for age 40+ female breast cancer cases, 2007-2017

|  | | **Odds of receiving CPM with reconstruction vs.** | | | | | |
| --- | --- | --- | --- | --- | --- | --- | --- |
|  |  | **CPM and no reconstruction** | | **non-CPM and no reconstruction** | | **UM and reconstruction** | |
|  |  | **OR** | **CI** | **OR** | **CI** | **OR** | **CI** |
| **Age at Diagnosis** | 40-49 years | **6.37** | **(5.95, 6.80)** | **25.64** | **(24.39, 27.03)** | **3.48** | **(3.26, 3.72)** |
|  | 50-59 years | **4.20** | **(3.94, 4.48)** | **10.75** | **(10.20, 11.36)** | **2.26** | **(2.11, 2.42)** |
|  | 60-69 years | **2.58** | **(2.43, 2.75)** | **4.93** | **(4.67, 5.18)** | **1.59** | **(1.49, 1.69)** |
|  | 70+ years | 1.00 | REF | 1.00 | REF | 1.00 | REF |
| **Race/Ethnicity** | American Indian | **0.67** | **(0.52, 0.86)** | **0.52** | **(0.43, 0.64)** | **0.67** | **(0.52, 0.87)** |
|  | Asian/Pacific Islander | **0.67** | **(0.63, 0.73)** | **0.46** | **(0.43, 0.48)** | **0.47** | **(0.44, 0.51)** |
|  | Black | **1.06** | **(1.01, 1.11)** | **0.55** | **(0.53, 0.57)** | **0.61** | **(0.59, 0.64)** |
|  | Hispanic | **1.42** | **(1.34, 1.51)** | **0.81** | **(0.78, 0.84)** | **0.73** | **(0.70, 0.77)** |
|  | Other/Unknown | 0.93 | (0.83, 1.04) | **0.77** | **(0.71, 0.83)** | **0.78** | **(0.71, 0.86)** |
|  | White | 1.00 | REF | 1.00 | REF | 1.00 | REF |
| **Insurance** | Not Insured | **0.42** | **(0.38, 0.47)** | **0.33** | **(0.30, 0.36)** | **0.71** | **(0.64, 0.79)** |
|  | Private Insurance | 1.00 | REF | 1.00 | REF | 1.00 | REF |
|  | Medicaid | **0.57** | **(0.54, 0.60)** | **0.54** | **(0.52, 0.56)** | **0.75** | **(0.72, 0.79)** |
|  | Medicare | **0.62** | **(0.60, 0.65)** | **0.60** | **(0.59, 0.63)** | **0.84** | **(0.81, 0.88)** |
|  | Other Government | **0.89** | **(0.80, 0.99)** | 0.94 | (0.87, 1.02) | 1.01 | (0.91, 1.12) |
|  | Insurance Status Unknown | **0.53** | **(0.47, 0.61)** | **0.30** | **(0.27, 0.33)** | **0.78** | **(0.69, 0.89)** |
| **Year of Diagnosis** | 2007-2009 | **0.61** | **(0.59, 0.63)** | **0.45** | **(0.44, 0.47)** | **0.53** | **(0.51, 0.55)** |
|  | 2010-2013 | **0.86** | **(0.83, 0.88)** | **0.87** | **(0.85, 0.88)** | **0.76** | **(0.74, 0.77)** |
|  | 2014-2017 | 1.00 | REF | 1.00 | REF | 1.00 | REF |
| **Median Income Quartiles**  **2008-2012** | <$38,000 | **0.48** | **(0.46, 0.51)** | **0.63** | **(0.61, 0.66)** | 1.04 | (0.99, 1.10) |
|  | $38,000-$47,999 | **0.58** | **(0.55, 0.60)** | **0.73** | **(0.71, 0.76)** | **1.07** | **(1.03, 1.11)** |
|  | $48,000-$62,999 | **0.68** | **(0.65, 0.70)** | **0.83** | **(0.81, 0.85)** | **1.06** | **(1.03, 1.09)** |
|  | >=$63,000 | 1.00 | REF | 1.00 | REF | 1.00 | REF |
| **Percent No High School Degree**  **Quartiles 2008-2012** | >=21% | 1.04 | (0.98, 1.09) | **0.81** | **(0.78, 0.84)** | **0.77** | **(0.73, 0.81)** |
|  | 13.0-20.9% | 1.00 | (0.96, 1.05) | **0.85** | **(0.83, 0.88)** | **0.82** | **(0.79, 0.86)** |
|  | 7.0-12.9% | **0.95** | **(0.92, 0.98)** | **0.90** | **(0.88, 0.92)** | **0.94** | **(0.91, 0.96)** |
|  | < 7.0% | 1.00 | REF | 1.00 | REF | 1.00 | REF |
| **Stage at Diagnosis** | 1 | 1.00 | REF | 1.00 | REF | 1.00 | REF |
|  | 2 | **0.74** | **(0.72, 0.76)** | **1.34** | **(1.32, 1.37)** | **0.84** | **(0.82, 0.86)** |
|  | 3 | **0.44** | **(0.43, 0.46)** | **1.38** | **(1.34, 1.42)** | **0.70** | **(0.67, 0.72)** |
| **Histology** | Invasive ductal carcinoma | 1.00 | REF | 1.00 | REF | 1.00 | REF |
|  | Invasive lobular carcinoma | **1.07** | **(1.04, 1.10)** | **1.49** | **(1.46, 1.52)** | 1.03 | (1.00, 1.06) |
|  | Other | **0.88** | **(0.82, 0.93)** | **0.87** | **(0.83, 0.91)** | **0.91** | **(0.86, 0.96)** |
| **Grade** | 1 | 1.00 | REF | 1.00 | REF | 1.00 | REF |
|  | 2 | **0.94** | **(0.91, 0.97)** | **1.18** | **(1.15, 1.20)** | 0.97 | (0.94, 1.00) |
|  | 3 | **0.83** | **(0.80, 0.87)** | **1.28** | **(1.25, 1.32)** | **1.16** | **(1.12, 1.20)** |
|  | 4 | **0.69** | **(0.54, 0.88)** | 1.14 | (0.93, 1.39) | 0.94 | (0.74, 1.21) |
|  | Unknown | **0.85** | **(0.80, 0.90)** | **1.33** | **(1.28, 1.39)** | 0.99 | (0.94, 1.05) |
| **Facility Type** | Community Cancer Program | 1.04 | (0.97, 1.10) | **0.90** | **(0.86, 0.95)** | 0.95 | (0.89, 1.02) |
|  | Comprehensive Community Cancer Program | 1.00 | REF | 1.00 | REF | 1.00 | REF |
|  | Academic/Research Program | 1.03 | (1.00, 1.06) | **0.92** | **(0.90, 0.94)** | **0.77** | **(0.75, 0.80)** |
|  | Integrated Network Cancer Program | **1.33** | **(1.28, 1.38)** | **1.28** | **(1.24, 1.31)** | 1.02 | (0.98, 1.05) |
| **Facility Region** | Midwest | **0.82** | **(0.78, 0.85)** | **1.22** | **(1.19, 1.25)** | **1.26** | **(1.22, 1.31)** |
|  | Northeast | 1.00 | REF | 1.00 | REF | 1.00 | REF |
|  | South | **0.72** | **(0.69, 0.75)** | **1.40** | **(1.37, 1.44)** | **1.52** | **(1.47, 1.56)** |
|  | West | **0.68** | **(0.65, 0.71)** | **1.20** | **(1.16, 1.23)** | **1.57** | **(1.51, 1.63)** |
| **Facility has reconstruction services** | Yes | 1.00 | REF | 1.00 | REF | 1.00 | REF |
|  | No | 0.001 | (0.001, 1000) | 0.001 | (0.001, 1000) | 1.40 | (0.001, 1000) |
| **Average annual surgical**  **volume 2007-2017** | <50 | **0.48** | **(0.45, 0.52)** | **0.50** | **(0.47, 0.53)** | **0.90** | **(0.83, 0.98)** |
|  | 50-99 | **0.55** | **(0.53, 0.58)** | **0.57** | **(0.55, 0.59)** | **0.89** | **(0.85, 0.93)** |
|  | 100-174 | **0.70** | **(0.68, 0.73)** | **0.81** | **(0.79, 0.83)** | 0.98 | (0.95, 1.01) |
|  | 175+ | 1.00 | REF | 1.00 | REF | 1.00 | REF |
| **Patient rurality crossed with**  **distance to surgery facility** | Metro patient who traveled <30 miles | 1.00 | REF | 1.00 | REF | 1.00 | REF |
|  | Non-metro bordering metro patient who traveled <30 miles | **0.74** | **(0.69, 0.79)** | **0.81** | **(0.76, 0.85)** | 1.07 | (1.00, 1.16) |
|  | Non-metro/rural patient who traveled <30 miles | **0.67** | **(0.59, 0.76)** | **0.77** | **(0.69, 0.85)** | **1.21** | **(1.04, 1.41)** |
|  | Metro patient who traveled 30-49 miles | 0.93 | (0.87, 1.00) | **1.23** | **(1.17, 1.31)** | **1.19** | **(1.10, 1.29)** |
|  | Non-metro bordering metro patient who traveled 30-49 miles | 0.94 | (0.80, 1.11) | **1.27** | **(1.11, 1.45)** | **1.30** | **(1.08, 1.56)** |
|  | Non-metro/rural patient who traveled 30-49 miles | **1.17** | **(1.10, 1.24)** | **1.43** | **(1.37, 1.49)** | **1.11** | **(1.05, 1.17)** |
|  | Metro patient who traveled 50+ miles | **1.21** | **(1.11, 1.31)** | **1.59** | **(1.50, 1.69)** | **1.11** | **(1.03, 1.20)** |
|  | Non-metro bordering metro patient who traveled 50+ miles | 1.04 | (0.96, 1.13) | **1.65** | **(1.56, 1.76)** | **1.24** | **(1.14, 1.34)** |
|  | Non-metro/rural patient who traveled 50+ miles | **1.28** | **(1.19, 1.37)** | **1.54** | **(1.46, 1.61)** | **1.12** | **(1.05, 1.19)** |
